# Heat Shock Protein 90 Family Isoforms as Prognostic Biomarkers and Their Correlations with Immune Infiltration in Breast Cancer

**DOI:** 10.1155/2020/2148253

**Published:** 2020-10-21

**Authors:** Tong Lin, Yuqin Qiu, Wenya Peng, Lisheng Peng

**Affiliations:** ^1^The Fourth Clinical Medical School, Guangzhou University of Chinese Medicine, Shenzhen 518033, China; ^2^Shenzhen Hospital of Traditional Chinese Medicine, Shenzhen 518033, China

## Abstract

**Background:**

The heat shock protein 90 (HSP90s) family is composed of molecular chaperones composed of four isoforms in humans, which has been widely reported as unregulated in various kinds of cancers. Nevertheless, the role of each HSP90s isoform in prognosis and immune infiltration in distinct subtypes of breast cancer (BRAC) remains unclear.

**Methods:**

Public online databases including the Oncomine, UALCAN, Kaplan-Meier Plotter, Tumor IMmune Estimation Resource (TIMER), Gene Expression Profiling Interactive Analysis (GEPIA), GeneMANIA, and Database for Annotation, Visualization, and Integrated Discovery (DAVID) were integrated to perform bioinformatic analyses and to explore the possible associations among HSP90s gene expression, prognosis, and immune infiltration in BRAC.

**Results:**

The mRNA expression of all HSP90s members was elevated in distinct clinical stages and subtypes of BRAC, compared with the normal breast tissue (*P* < 0.05). Overexpressed HSP90AA1 was associated with poor prognosis, particularly, both short overall survival (OS) and release-free survival (RFS) in Basal-like BRAC patients; overexpressed HSP90AB1 and HSP90B1 were both associated with poor RFS in Luminal A BRAC patients, while overexpressed TRAP1 was associated with favorable RFS in Luminal A BRAC patients. Moreover, HSP90s gene expression in BRAC showed correlations with the infiltration of CD8+ T cells, neutrophils, macrophages, and dendritic cells (DCs), as well as the activation of tumor-associated macrophages (TAMs), DCs, and CD4+ helper T (Th) cells. The underlying mechanisms of HSP90s modulating tumor-infiltrating immune cells (TIICs) might be related with their functions in antigen processing and presentation, major histocompatibility complex (MHC) binding, and assisting client proteins.

**Conclusion:**

This study demonstrated that HSP90s family genes were overexpressed and might be serve as prognostic biomarkers in subtypes of BRAC. It might be a novel breakthrough point of BRAC treatment to regulate immune infiltration in BRAC microenvironment for more effective anticancer immunity through pharmacological intervention of HSP90s.

## 1. Introduction

Despite that mortality has declined by improvements in screening and adjuvant systemic treatments, breast cancer (BRAC) is still the most common cancer and the leading cause of cancer-related death for females worldwide [1, 2]. BRAC is a highly heterogenous disease in histology and molecular, determining different incidence, biology, treatment sensitiveness, and prognosis [3, 4]. According to the distinct expression of molecular signatures, including estrogen receptor (ER), progesterone receptor (PR), and human epidermal growth factor receptor 2 (HER2), BRAC is mainly classified into four subtypes: Luminal A (ER+ and/or PR+ and HER2−), Luminal B (ER+ and/or PR+ and HER2+ or HER2−), HER2-enriched (ER−, PR−, and HER2+), and Basal-like (ER−, PR−, and HER2−). Basal-like BRAC were thought to be interchangeable with Triple-Negative BRAC (TNBC) in the past. Nevertheless, TNBC is now widely acknowledged as a heterogeneous disease itself, although the Basal-like subtype accounts for 80.6% of it [5].

Tumor-infiltrating immune cells (TIICs) in the tumor microenvironment (TME) play critical roles in initiation, progression, metastasis, and treatment resistance of the tumor [4]. Distinctive subsets of TIICs can act oppositely. For example, CD8+ T cells and M1 macrophages exert cytotoxic immune surveillance to inhibit cancer growth, while CD4+ regulatory T (Treg) cells and M2 macrophages suppress effective anticancer immunity [6, 7]. Notwithstanding the paradoxical roles of subsets of TIICs, increased density of tumor-infiltrating lymphocytes is now accepted as an indicator of better treatment responses and favorable outcomes in BRAC patients, particularly TNBC and HER2-enriched BRAC [8–10]. Although the development of immunotherapy has revolutionized the area of cancer, the clinical efficacy is still limited [6]. In this case, a deeper understanding of the nature of tumor immunity in BRAC can help to empower new treatment strategies.

The heat shock protein 90 (HSP90s) family is composed of ubiquitously ATP-dependent molecular chaperones assisting in folding newly synthesized proteins or stabilizing denatured proteins under stress [11, 12]. The client proteins of HSP90s include receptor tyrosine kinases, transcription factors, steroid hormone receptors, and cell cycle regulatory proteins, all of which play essential roles in cell survival and proliferation, as well as oncogenesis and malignancy of tumor [13]. Considering the vital functions of HSP90s, it is not surprising that they are reported as highly expressed and could serve as unfavorable prognostic biomarkers in various kinds of human cancers, including BRAC [14–16]. The human HSP90s family has four isoforms, HSP90*α* and HSP90*β* locate in the cytoplasm and GRP94 and TRAP1 (also TNF receptor-associated protein 1) locate in the endoplasmic reticulum and mitochondria, respectively. These proteins are encoded by four real genes in humans, HSP90AA1, HSP90AB1, HSP90B1, and TRAP1 [17].

The diagnostic, prognostic, or predictive potentials of HSP90s genes had been partly reported previously [3, 14–18]. However, the role of each HSP90s family member in the development and progression of every certain subtype of BRAC remains unknown. What is more, HSP90s are reported to modulate immune processes, such as antigen presentation and activation of lymphocyte [19, 20]. However, whether HSP90s is involved in the regulations of immune infiltration has not been studied yet. In this study, we comprehensively analyzed the expression profiles and prognostic values of HSP90s family genes in different subtypes of BRAC, and their correlations with TIICs were evaluated to explore the possible mechanisms by which HSP90s affects the progression of BRAC, integrating several publicly available databases. The findings of this study should advance our understanding of associations among HSP90s gene expression, survival outcomes, and immune infiltration in BRAC, which might provide novel insights for treatment strategy.

## 2. Materials and Methods

### 2.1. Analysis of HSP90s Gene Expression

The Oncomine is an online data mining server unifying a large compendium of microarray data across 18,000 cancer samples, combined the published literature, the Stanford Microarray Database and the NCBI Gene Expression Omnibus (GEO) [21]. The Oncomine was used to perform a meta-analysis of mRNA expression of HSP90s in various types of cancers. Transcriptional expression levels of HSP90s in cancer and normal tissues were compared using Students' *t*-test. The threshold was set as *P* = 0.01, a fold change of 2.0, and top 10% gene ranking.

The UALCAN database is an interactive web portal for in-depth gene expression analysis using RNA sequencing (RNA-seq) and clinical data from the Cancer Genome Atlas (TCGA) project [22]. The UALCAN was used to evaluate the expression of HSP90s genes in all BRAC and distinct clinicopathological stages and subtypes of BRAC, compared with the normal breast tissues.

### 2.2. Analysis of Prognostic Significance of HSP90s Genes in BRAC Patients

The Kaplan-Meier (KM) Plotter is an online database that provides 54,000 gene expression profiles with patients' survival information in twenty-one kinds of cancers, which harbors gene chip and RNA-seq data from GEO, TCGA, and European Genome-phenome Archive [23]. KM Plotter was applied to assess the associations between HSP90s gene expression and survival of BRAC, using univariate analysis. All cases were categorized into high and low expression groups by the median expression of a certain gene. Survival curves, hazard ratio (HR), 95% confidence intervals (CI), and log-rank *P* values were generated online. *P* < 0.05 was considered to be statistically significant.

### 2.3. Associations between HSP90s Gene Expression and Immune Infiltration in BRAC

The Tumor IMmune Estimation Resource (TIMER) is a website for the investigation of tumor immune interactions, which incorporates 10,897 samples from thirty-two kinds of cancers from the TCGA [24]. The correlations between HSP90s gene expression and the infiltration levels of six kinds of TIICs (B cells, CD4+ T cells, CD8+ T cells, neutrophils, macrophages, and dendritic cells (DCs)) in all BRAC and distinct subtypes of BRAC were evaluated using the TIMER.

Gene Expression Profiling Interactive Analysis (GEPIA) is a database used to analyze RNA-seq data, based on 9,736 tumors and 8,587 normal samples from the TCGA and the GTEx projects [25]. Correlations between HSP90s gene expression and gene biomarkers of TIICs were further investigated, combining the TIMER and GEPIA databases. The correlation coefficient in both databases was analyzed by the Spearman method, and *P* < 0.05 was considered statistically significant. The correlation strength was evaluated by the values of the correlation coefficient, according to the previous studies: 0.00–0.19 “very weak,” 0.20–0.39 “weak,” 0.40–0.59 “moderate,” 0.60–0.79 “strong,” and 0.80–1.0 “very strong” [26, 27].

### 2.4. Gene Interaction Network of HSP90s and Functional Enrichment Analysis

GeneMANIA is an online tool for investigation into associated or similar genes for target genes, through analysis of physical and functional associations, such as colocalization, coexpression, and physical interaction [28]. We constructed the gene interaction network of HSP90s using the GeneMANIA. All genes in the interaction network were then imported to Database for Annotation, Visualization, and Integrated Discovery (DAVID) server for Gene Ontology (GO) and Kyoto Encyclopedia of Genes and Genomes (KEGG) pathway enrichment analyses. GO enrichment analysis predicted the functions of genes in three aspects, including biological process (BP), cellular component (CC), and molecular function (MF). *P* < 0.05 and false discovery rate (FDR) < 0.05 were considered as statistically significant.

## 3. Results

### 3.1. Overexpression of HSP90s Genes in Various Kinds of Cancers and BRAC

**Figure 1:**
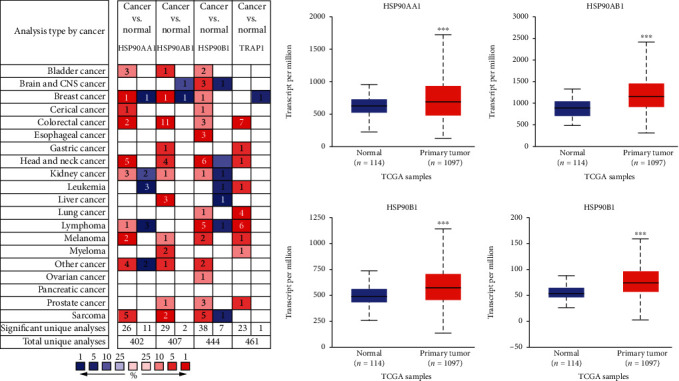
Transcriptional expression of HSP90s isoforms in various kinds of human cancers and BRAC. (a) Transcriptional expression of HSP90s isoforms in various kinds of cancers (the Oncomine). Notes: number in the colored cell is equal to the number of datasets with statistically significant HSP90s mRNA up expression (red) or down expression (blue), compared with the corresponding normal samples. Color depth represents a median rank of a gene, across all the included analyses. (b) Transcriptional expression of HSP90s isoforms in BRAC, compared with the normal breast tissues (the UALCAN) (^∗^*P* < 0.05, ^∗∗^*P* < 0.01, and ^∗∗∗^*P* < 0.001).

First, the differential expression of HSP90s genes in various kinds of cancers and the corresponding normal samples were analyzed using the Oncomine database. As shown in Figure 1(a), elevated mRNA expression of HSP90s was observed in many kinds of cancers, except for pancreatic cancer. HSP90AA1, HSP90AB1, and HSP90B1 each were significantly overexpressed in one dataset of BRAC, while no significant differential expression of TRAP1 was observed, with the above threshold (Table 1). It was notable that none of the HSP90s family gene was highly expressed in any kind of normal tissue in any dataset.

Thereafter, the differences of HSP90s gene expression between all BRAC samples and normal breast samples were verified using the UALCAN database. As shown in Figure 1(b), the expression levels of all HSP90s family genes were significantly higher in BRAC samples than in normal breast samples (*P* < 0.001).

### 3.2. Expression of HSP90s Genes in Distinct Clinicopathological Stages and Intrinsic Subtypes of BRAC

**Figure 2:**
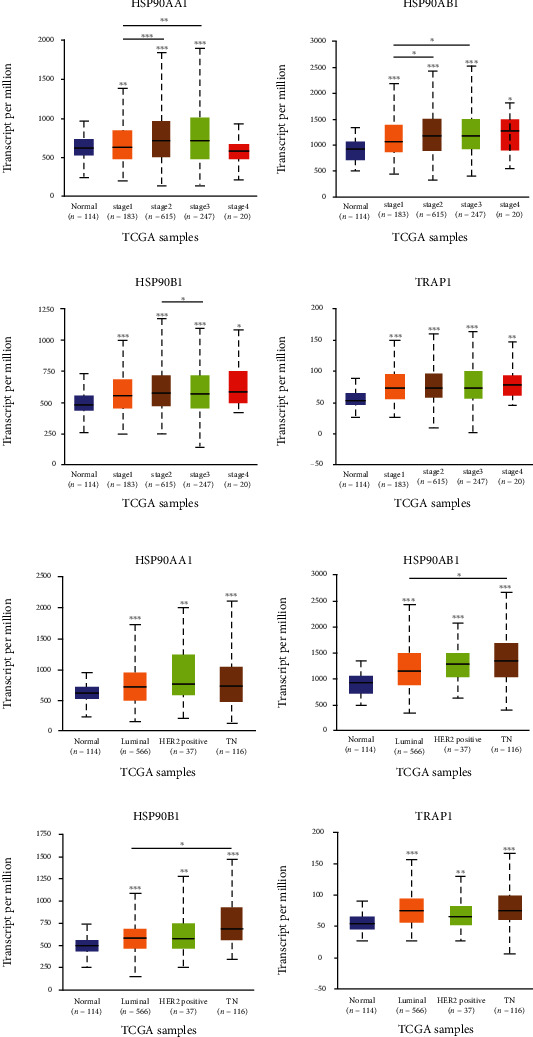
Expression of HSP90s genes in BRAC by clinicopathological stages and intrinsic subtypes (the UALCAN). Expression of HSP90AA1, HSP90AB1, HSP90B1, and TRAP1 in BRAC classified by (a) clinicopathological stages and (b) intrinsic subtypes (^∗^*P* < 0.05, ^∗∗^*P* < 0.01, and ^∗∗∗^*P* < 0.001).

Next, the expression of HSP90s genes in distinct clinicopathological stages and intrinsic subtypes of BRAC was analyzed using the UALCAN database. It was shown that every member of HSP90s genes was highly expressed in every clinicopathological stage of BRAC, compared with normal tissues, except for HSP90AA1 in the fourth stage of BRAC (*P* < 0.05, Figure 2(a)). The expression levels of HSP90AA1 and HSP90AB1 were both significantly higher in the second and third stages of BRAC, compared with those in the first stage (*P* < 0.05).

In respect of intrinsic subtypes, all members of the HSP90s genes family were highly expressed in four subtypes of BRAC, compared with the normal tissues (*P* < 0.01, Figure 2(b)). Besides that, the expression levels of HSP90AB1 and HSP90B1 were both significantly higher in TNBC, compared with those in Luminal-like BRAC (*P* < 0.05).

### 3.3. Prognostic Significance of HSP90s Genes in All BRAC

**Figure 3:**
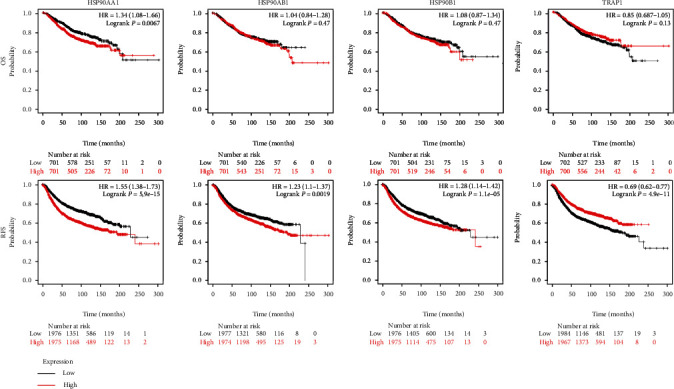
Prognostic significance of HSP90s genes in all BRAC patients (the KM Plotter). The Kaplan-Meier plots showed the associations between the HSP90s gene expression with OS and RFS in all BRAC patients. Note: Affymetrix IDs for HSP90AA1, HSP90AB1, HSP90B1, and TRAP1 were “214328_s_at,” “200064_at,” “200599_s_at,” and “221235_s_at,” respectively. *P* < 0.05 was thought as significant. Abbreviations: OS: overall survival; RFS: relapse-free survival; HR: hazard ratio.

We had discovered that HSP90s genes were consistently significantly highly expressed in BRAC. Then, the prognostic significance of HSP90s genes in all BRAC patients was analyzed using the KM Plotter database. As shown in Figure 3, BRAC patients with high HSP90AA1 expression had both shorter overall survival (OS) (*P* = 0.0067) and relapse-free survival (RFS) (*P* = 5.9*E*‐15); patients with high expression of HSP90AB1 and HSP90B1 both had shorter RFS (*P* = 1.9*E*‐4 and *P* = 1.1*E*‐5, respectively), while patients with high expression of TRAP1 had longer RFS (*P* = 4.9*E*‐11).

### 3.4. Prognostic Significance of HSP90s Genes in BRAC with Distinct Clinical Parameters

**Figure 4:**
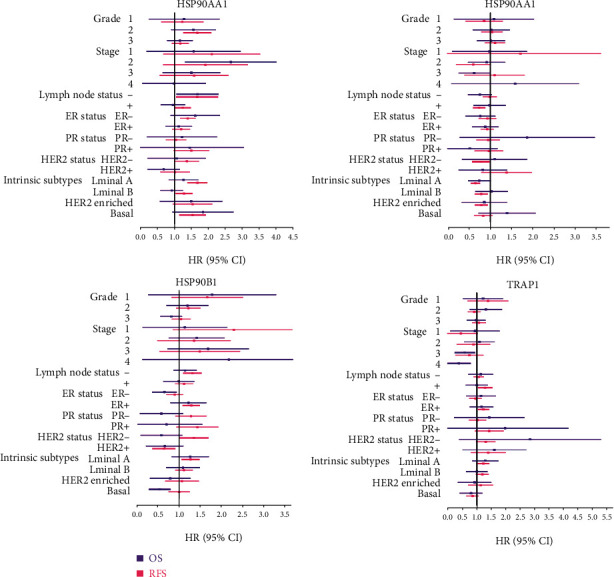
Prognostic significance of HSP90s genes in BRAC patients with different clinical parameters. Note: forest plots showed HR and 95% CI of the univariate survival analyses; the results with statistical significance are in bold. The data of RFS in the fourth stage of BRAC patients could not be analyzed due to the insufficient sample size. Abbreviations: OS: overall survival; RFS: relapse-free survival; HR: hazard ratio; CI: confidence interval.

Since the survival outcomes of BRAC patients largely differ with the clinicopathological characteristics, we access the prognostic values of HSP90s genes in BRAC patients with distinct clinical parameters. We found that high HSP90AA1 expression was associated with both poorer OS and RFS of the Basal-like subtype (OS: HR = 1.69, *P* = 0.038; RFS: HR = 1.51, *P* = 0.0013) and negative lymph node status (OS: HR = 1.6, *P* = 0.013; RFS: HR = 1.6, *P* = 0.013) of BRAC patients; poorer OS of the second stage (HR = 2.43, *P* = 5.70*E*‐4) of BRAC patients; and poorer RFS of ER+ (HR = 1.39, *P* = 6.80*E*‐05), PR+ (HR = 1.45, *P* = 0.035), HER2− (HR = 1.35, *P* = 0.027), Luminal A (HR = 1.67, *P* = 4.80*E*‐09), Luminal B (HR = 1.28, *P* = 0.013), HER2-enriched (HR = 1.47, *P* = 0.049), and positive lymph node status (HR = 1.24, *P* = 0.03), as well as the second grade (HR = 1.65, *P* = 5.10*E*‐05) of BRAC patients (Figure 4 and Supplementary Tables 1, 2, 3, and 4).

High HSP90AB1 expression was associated with poorer RFS of ER+ (HR = 1.29, *P* = 0.002), HER2− (HR = 1.33, *P* = 0.031), Luminal A (HR = 1.27, *P* = 0.006), and negative lymph node status (HR = 1.32, *P* = 0.001) of BRAC patients, while better OS of ER− (HR = 0.61, *P* = 0.033) and the Basal-like subtype (HR = 0.5, *P* = 0.005) of BRAC patients, and better RFS of HER2+ (HR = 0.61, *P* = 0.024) of BRAC patients. High HSP90B1 expression was associated with poorer RFS of ER+ (HR = 1.24, *P* = 0.009), Luminal A (HR = 1.24, *P* = 0.015), and positive lymph node positive status (HR = 1.29, *P* = 0.012) of BRAC patients, while better OS of the third stage (HR = 0.54, *P* = 0.043) and the fourth stage (HR = 0.26, *P* = 0.020) of BRAC patients. High TRAP1 expression was associated with better RFS of HER2− (HR = 0.77, *P* = 0.046), Luminal A (HR = 0.66, *P* = 1.90*E*‐06), and positive lymph node status (HR = 0.74, *P* = 0.002) of BRAC patients.

Taken together, the findings suggested that highly expressed HSP90AA1 was associated with poor prognosis of BRAC patients, independent of intrinsic subtype and lymph node status. Highly expressed TRAP1 indicated a favorable prognosis in Luminal A and lymph node metastatic BRAC patients. HSP90AB1 and HSP90B1 seemed to perform dual effects on the prognosis of BRAC patients; high expression of both of them was associated with unfavorable outcomes of ER+ and Luminal A patients. Meanwhile, highly expressed HSP90AB1 was also linked with favorable outcomes in ER−, Basal-like, and HER2+ patients, and highly expressed HSP90B1 was linked with better outcomes of the advanced patients.

### 3.5. Correlations between HSP90s Gene Expression and Immune Infiltration in BRAC

**Figure 5:**
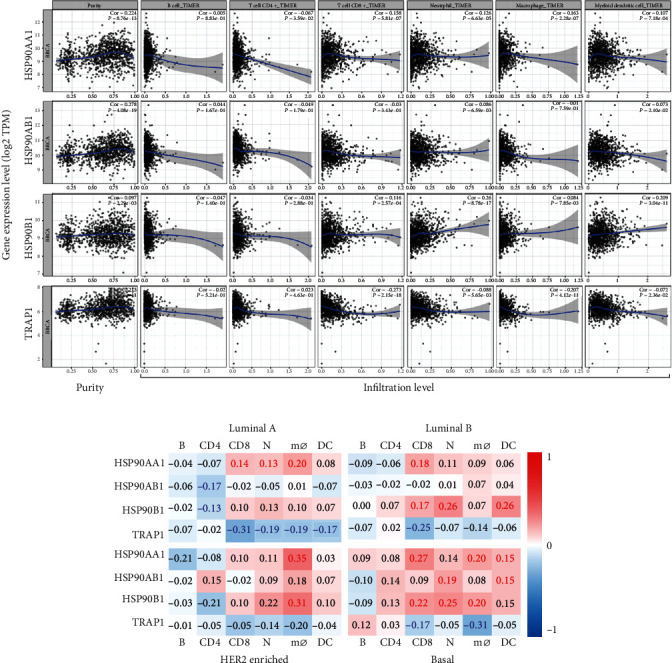
Correlations of HSP90s gene expression with immune infiltration in BRCA. (a) Correlations of HSP90s gene expression with tumor purity and infiltration of B cells, CD8+ T cells, CD4+ T cells, macrophages, neutrophils, and DCs in all BRCA (the TIMER). (b) Heat maps show the correlations of HSP90s gene expression with immune filtration in subtypes of BRCA. Note: correlation coefficients with statistical significance are shown in bold; red or blue color represents positive or negative correlations, respectively. Abbreviations: B: B cells; CD4: CD4+ T cells; CD8: CD8+ T cells; N: neutrophils; mø: macrophages; DC: dendritic cells.

Correlations between HSP90s gene expression and immune infiltration in BRAC were investigated using the TIMER server. Because the evaluation of immune infiltration is influenced by tumor purity, which means the proportion of cancer cells in the admixture [32], the correlation analysis was adjusted for corresponding tumor purity. The results showed that the expression of HSP90AA1, HSP90AB1, and TRAP1 was positively correlated to the tumor purity, while that of HSP90B1 was not (Figure 5(a)). The expressions of HSP90AA1 and HSP90B1 were both positively correlated with the infiltration of CD8+ T cells, neutrophils, macrophages, and DCs (except for HSP90B1 with macrophages), while the expression of TRAP1 was negatively correlated with the infiltration of CD8+ T cells and macrophages, though the correlation strengths were all weak.

Since the density, subpopulations, and activity of TIICs could lead to distinct outcomes and therapeutic responses of different subtypes of BRAC [33, 34], relations between HSP90s gene expression and immune infiltration in different subtypes of BRCA were analyzed as well. The findings could be roughly described in that HSP90AA1 expression had positive correlations with the infiltration of CD8+ T cells, neutrophils, and macrophages in Luminal A, and CD8+ T cells, macrophages, and DCs in Basal-like BRAC. HSP90AB1 had positive correlations with the infiltration of neutrophils and DCs in Basal-like BRAC. HSP90B1 had positive correlations with the infiltration of CD8+ T cells, neutrophils, and DCs in Luminal B, and CD8+ T cells, neutrophils, and macrophages in Basal-like BRAC. Besides that, the expression of HSP90AB1 and HSP90B1 was negatively correlated with the infiltration of CD4+ T cells in Luminal A BRAC. TRAP1 had negative correlations with CD8+ T cells, neutrophils, macrophages, and DCs in Luminal A and CD8+ T cells and macrophages in Basal-like BRAC. It could be noted that HSP90s showed the most correlations with immune infiltration in Basal-like BRAC.

### 3.6. Correlations between HSP90s Gene Expression and Biomarkers' Expression of Subsets of TIICs in BRAC

To further explore the activation status of TIICs, the correlations between the HSP90s gene expression and biomarkers' expression of subsets of TIICs were analyzed, combined with the TIMER and GEPIA databases. There were some differences between the results from the two databases; here, we only expounded on the coincident results (Tables 2 and 3). In BRAC tissues, the expression of HSP90AA1, HSP90AB1, and HSP90B1 was conformably correlated with the expression of biomarkers of some lineages of CD4+ helper T (Th) cells and tumor-associated macrophages (TAMs). Namely, they were significantly positively correlated with the expression of STAT1 (Th1), IL21 (Tfh), STAT3 (Th17), CCR8 (Treg), CD68, and IL10 (TAM), while they were also negatively correlated with the expression of STAT5A (Th2), TGFB1 (Treg), HLA-DPB1, HLA-DQB1, and CD1C (DC). The HSP90B1 expression in BRAC was also significantly positively correlated with TNF and IFNG (Th1), TIM3 (T cell exhaustion), KIR2DL1 and KIR3DL3 (NK cell), IRF5 (M1 macrophage), and CD163 and MS4A4A (M2 macrophage), whereas negatively correlated with the expression of GATA3 (Th2). TRAP1 expression in BRAC was significantly positively correlated with the expression of TNF (Th1) and STAT6 (Th2) and negatively correlated with the expression of STAT4 (Th1), PEGS2 (M1 macrophage), MS4A4A (M2 macrophage), CCL2 (TAM), HLA-DPA1, CD1C, and NRP1 (DC).

Generally speaking, we found that the HSP90s gene expression was correlated with the expression of biomarkers of lineages of Th (Th1, 2, 17, Treg, and Tfh) cells, TAM, and DCs. Even though the infiltration of CD8+ T cells and neutrophils was positively correlated with the expression of HSP90AA1 and HSP90B1, the expression of their biomarkers showed no significant correlation with the HSP90s gene expression based on the TIMER database, while negative correlations based on the GEPIA database, which requires further explorations. Even so, all the above findings stated that HSP90s genes might partly modulate the infiltration and activation of TIICs in BRAC.

### 3.7. Functions of Gene Interaction Network of HSP90s

**Figure 6:**
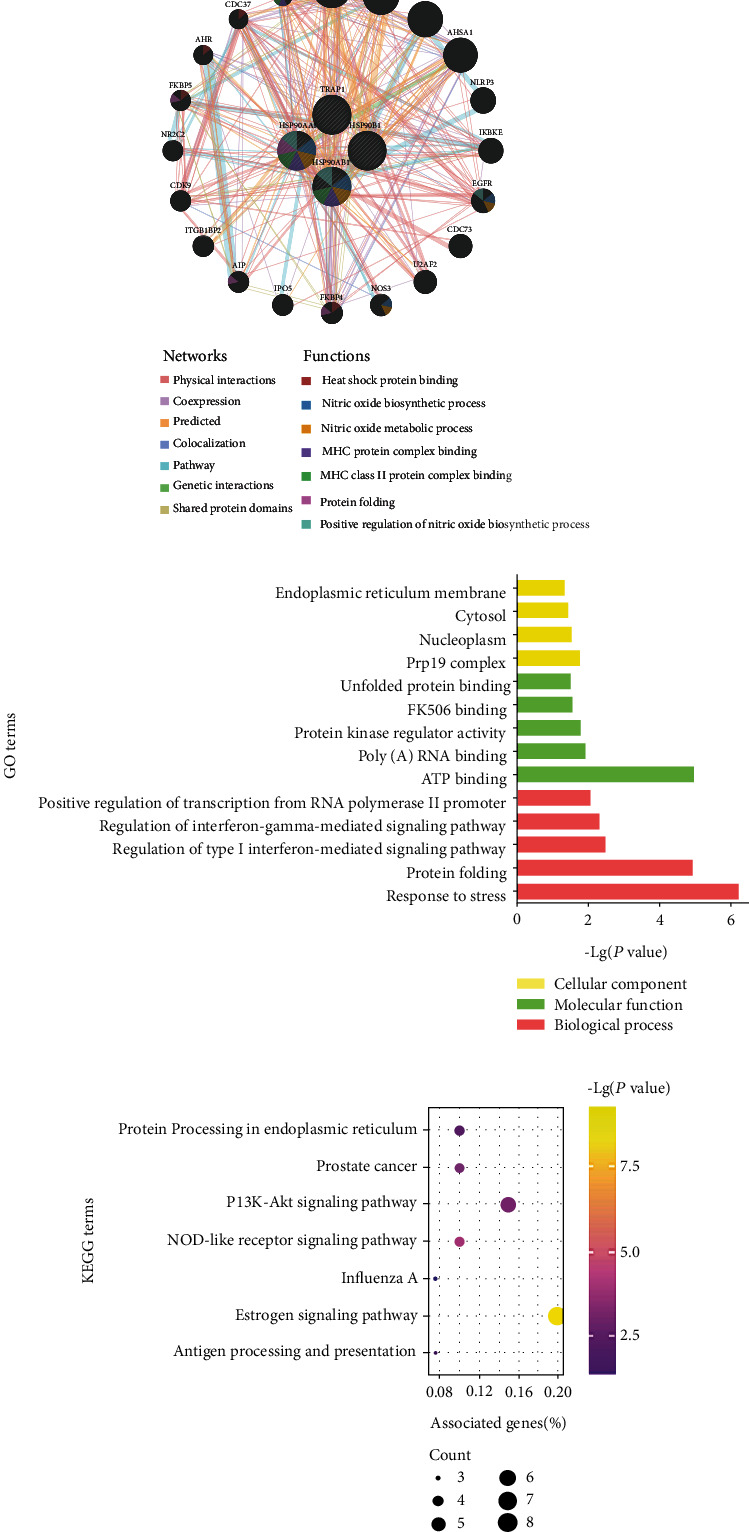
Functions of HSP90s interaction network. (a) The gene interaction network of HSP90s constructed by the GeneMANIA. Note: distinct colors of the network edges represent the distinct bioinformatic methods applied: physical interactions, coexpression, predicted, colocalization, pathway, genetic interactions, and shared protein domains. Distinct colors of the network nodes represent the biological functions of the sets of genes. (b) The results of GO enrichment analysis: the top five terms of BP and MF and all terms of CC are shown. (c) The results of KEGG enrichment analysis: all KEGG terms are shown.

To understand the biological functions of HSP90s, a gene interaction network was constructed using the GeneMANIA. Twenty HSP90s-associated genes were observed in the interaction network, functions of which focused on heat shock protein binding, nitric oxide biosynthetic and metabolic process, major histocompatibility complex (MHC) protein complex binding, and protein folding (Figure 6(a)).

Enrichment analyses were conducted to further investigate the potential biological functions of the twenty-four interactive genes using the DAVID database. The five most significantly enriched GO-BP and GO-MF terms and all significantly enriched GO-CC terms are shown in Figure 6(b), which elucidated that the cellular response to stress, protein folding, interferon-mediated signaling pathway, and transcription progress were regulated by the HSP90s interaction network. Besides, seven KEGG terms were significantly enriched, suggesting that the signaling pathways of estrogen, PI3K-Akt, and NOD-like receptor, as well as biological processes of antigen processing and presentation, were related (Figure 6(c)).

## 4. Discussion

Cancer cells rely on HSP90s to support the activation, mutation, translocation, or overexpression of multiple oncoproteins. Thus, cancer cells are usually addicted to HSP90s to relieve the intracellular stress caused by their malignant lifestyle, which consequently facilitates cancer progression and treatment resistance [35, 36]. In this context, HSP90s are frequently observed overregulated in a wide range of cancers, and numerous HSP90s inhibitors are under trial as promising agents for cancer [11]. In this study, we found that all members of the HSP90s gene family were significantly overexpressed in many kinds of cancers, including BRAC (Figure 1), and they were consistently elevated in different clinical stages and subtypes of BRAC (Figure 2), compared with the corresponding normal tissues.

Previous studies mostly demonstrated that high HSP90s expression was relevant with unfavorable prognosis or treatment response. A research illuminated that HSP90AA1 was overexpressed in BRAC and was correlated with shorter OS and aggressive clinicopathological features, including high clinical stage, large tumors, and lymph node involvement [37]. Jarzab et al. found that low expression of HSP90AA1 and HSP90AB1 might indicate higher sensitivity to chemotherapy and higher probability of pathological complete response in BRAC patients [18]. Cheng et al. found that high expression of HSP90AA1 and HSP90AB1 might be independent factors predicting poor prognosis of TNBC and ER+/HER2− patients, respectively, which was quite inconsistent with our findings [16]. Elevated protein expression of HSP90B1 was reported to be closely linked to the progression, distant metastasis, and decreased OS in BRAC patients [38, 39]. And TRAP1 was described to be involved in energetic metabolism in various cancer cells and was often associated with treatment resistance, but the exact role of which remains controversial [14].

In the current study, we had some new findings about the prognostic values of HSP90s genes and their associations with immune infiltration in BRAC. Our results suggested that high HSP90AA1 expression indicated poor prognosis in BRAC patients, independent of intrinsic subtypes or lymph node status. In particular, it was linked with both worse OS and RFS in Basal-like BRAC patients (Figure 4). Additionally, highly expressed HSP90AA1 showed positive correlations with the infiltration of CD8+ T cells, neutrophils, macrophages, and DCs in Luminal A and Basal-like BRAC (Figure 5(b)). Highly expressed HSP90AB1 and HSP90B1 were both linked with unfavorable RFS in Luminal A BRAC patients, with negative correlations with the infiltration of CD4+ T cells, whereas TRAP1 overexpression implied favored outcomes in Luminal A BRAC patients, with negative correlations with CD8+ T cells, neutrophils, macrophages, and DCs. Furthermore, the expression of HSP90s family genes showed correlations with the expression of biomarkers of Th (Th1, 2, 17, Treg, and Tfh) cells, TAMs, and DCs, indicating their participation in the activation and recruitment of TIICs in BRAC. Hereto, we could summarize that high expression of HSP90s family genes could serve as prognostic biomarkers of BRAC, especially of Basal-like and Luminal A BRAC. And the infiltration of CD8+ T cells, neutrophils, macrophages, and DCs, along with the activation of Th cells, TAMs, and DCs, might be involved.

CD8+ T cells are the key undertakers of anticancer immunity. Once stimulated by cancer antigens and cytokines secreted by Th1 cells, they further proliferate and differentiate into effective cytotoxic cells with specific cancer-killing capabilities [10, 40, 41]. The role of tumor-associated neutrophils in BRAC is still unclear, but some preclinical studies identified their immunosuppression by reducing T cell proliferation [42]. TAMs may constitute over 50% of the number of cells within TME, which are classified into classically activated M1 and alternatively activated M2 subtypes. M1 macrophages can be stimulated by Th1 cytokines, then release proinflammatory cytokines to enhance anticancer immunity. Inversely, M2 macrophages can be stimulated by Th2 cytokines, then produce anti-inflammatory cytokines to hinder effective immunity. Generally, the high density of TAMs predicts unfavorable survival; either depletion of TAMs or reversion of M2 to M1 has been reported to inhibit cancer progression in mouse models of BRAC [43–45]. DCs are antigen-presenting cells (APCs) specialized in triggering *de novo* T cell responses, and they also maintain the response effectiveness within the peripheral tissues [46, 47].

Naive CD4+ T cells can differentiate into several lineages of Th cells, including Th1, Th2, Th17, Treg, and Tfh, distinguished by their patterns of cytokine production and biological functions [48]. Th1 cells can activate CD8+ T cells and enhance anticancer immunity by secreting interferon-*γ* and interleukin (IL) 2, while Th2 cells express IL4, IL5, IL6, IL10, and IL13 that induce anergy of T cells. Thus, a higher value of Th1/Th2 is an indicator of better outcomes in cancer patients [49]. Th17 cells have exceptional roles in the development and progression of BRAC, which sustain procancer chronic inflammation through production of proinflammatory cytokines [50]. Treg cells, especially the FOXP3+ ones, suppress immune responses and maintain cancer immune tolerance, the frequency of which has been applied as an independent risk factor of cancer relapse [51, 52]. Tfh cells could organize immune structures adjacent to the tumor bed which potentially propagates sustainable anticancer immunity, so the signatures of Tfh cells could be predictors of better postsurgical outcomes [53].

Based on the discussions above, it could be concluded that the HSP90s-associated TIICs in BRAC work both in immunostimulatory and immunosuppressive manners, which was an echo of the paradoxical character of the HSP90s family in tumor immunity. The enrichment analyses revealed that HSP90s function as immune regulators in antigen processing and presentation, MHC binding, and the interferon-mediated signaling pathway. In fact, HSP90s serve as immunogens themselves. HSP90s exposed on the surfaces of dying cancer cells are kinds of “danger signals” to prompt APC activation and consequently stimulate effector T cells [54]. Despite the immunostimulatory roles of HSP90s, abundance of evidence suggests that the HSP90s blockade could potentiate anticancer immunity and support combination with immunotherapies [20]. There are primarily two potential mechanisms. On the one hand, HSP90s inhibition could increase the expression of tumor-specific antigens and MHC I-complexed antigens, which synergistically potentiate immunogenicity, thus enhancing tumor surveillance [55, 56]. On the other hand, client proteins that may drive the checkpoint programmed cell death protein 1 (PD-1) and its ligand (PD-L1) expression, such as mutant EGFR, JAK2, and HIF1*α*, would be under destabilization once HSP90s are inhibited. Loss of PD-1/PD-L1 expression would consequently cut down the activities of immune checkpoints and restore T cell-mediated cytotoxicity [19]. In short, based on the evidence so far, the combination of immunotherapies and HSP90s inhibitors appears to be a promising therapeutic strategy. Moreover, we propose that regulating TIICs to enhance the anticancer immunity in BRAC through HSP90s intervention might be a new breakthrough point of treatment, but more verifications are required.

Last but not least, there were some unexpected findings in our study. We found that high expression of HSP90AB1 and HSP90B1 was both associated with better outcomes in some specific types of BRAC patients. Therefore, these contradictory effects of HSP90s genes need more explorations.

## 5. Conclusions

In conclusion, we discovered that HSP90s family isoforms were all overexpressed in BRAC. Elevated HSP90AA1, HSP90AB1, and HSP90B1 expression might serve as unfavorable prognostic biomarkers of BRAC, while TRAP1 might act as a favorable one. HSP90s might modulate the TME in BRAC through regulating infiltration of CD8+ T cells, neutrophils, macrophages, and DCs, as well as activation of Th, TAM, and DC cells. The underlying mechanisms might be related to HSP90s' functions in antigen processing and presentation, MHC binding, and assisting client proteins. This study suggested HSP90s as potential therapeutic targets to modulate anticancer immunity, and the combination of immunotherapies and HSP90s inhibitors might be a promising treatment strategy. However, further investigations are still necessary.

## Figures and Tables

**Table 1:** The significant differential expression of HSP90s genes between BRAC and normal samples from the Oncomine.

Gene name	Types of breast cancer vs. breast	Fold change	*P* value	*t*-test	Reference
HSP90AA1	Ductal breast carcinoma vs. normal	2.225	2.76*E*-09	8.84	[29]
HSP90AB1	Mucinous breast carcinoma vs. normal	2.203	1.68*E*-09	12.035	[30]
HSP90B1	Lobular breast carcinoma vs. normal	2.172	5.67*E*-04	6.373	[31]

**Table 2:** Correlations between HSP90s gene expression and biomarker expression of subsets of TIICs in BRAC from the TIMER.

Types of TIICs	Gene markers	HSP90AA1		HSP90AB1		HSP90B1		TRAP1	
		*R*	*P*	*R*	*P*	*R*	*P*	*R*	*P*
B cell	CD19	-0.02	4.90*E*-01	-0.01	7.71*E*-01	0.01	8.13*E*-01	0.04	2.19*E*-01
	CD79A	-0.04	1.81*E*-01	0.00	9.59*E*-01	0.00	9.37*E*-01	0.02	5.76*E*-01

T cell (general)	CD3D	-0.08	**1.53** **E** **-02**	-0.03	3.43*E*-01	0.02	4.88*E*-01	-0.01	6.66*E*-01
	CD2	0.02	5.01*E*-01	0.04	1.90*E*-01	0.08	**1.62** **E** **-02**	-0.02	5.87*E*-01

Th1	TBX21	-0.05	1.42*E*-01	0.02	5.04*E*-01	0.06	**4.77** **E** **-02**	0.02	6.08*E*-01
	STAT4	0.06	7.49*E*-02	0.01	7.21*E*-01	0.08	**7.90** **E** **-03**	-0.10	**2.04** **E** **-03**
	STAT1	0.33	**1.08** **E** **-26**	0.29	**1.25** **E** **-20**	0.34	**3.99** **E** **-28**	0.03	3.26*E*-01
	TNF	0.03	3.79*E*-01	0.08	**9.84** **E** **-03**	0.13	**4.54** **E** **-05**	0.16	**6.65** **E** **-07**
	IFNG	0.08	**1.17** **E** **-02**	0.12	**2.14** **E** **-04**	0.20	**2.28** **E** **-10**	0.01	7.89*E*-01

Th2	GATA3	0.02	5.29*E*-01	-0.04	2.66*E*-01	-0.20	**3.90** **E** **-10**	0.02	6.23*E*-01
	STAT6	0.01	7.19*E*-01	-0.05	1.31*E*-01	-0.12	**2.52** **E** **-04**	0.07	**2.13** **E** **-02**
	IL13	-0.02	5.17*E*-01	0.02	5.00*E*-01	0.03	2.99*E*-01	-0.02	5.44*E*-01
	STAT5A	-0.16	**2.45** **E** **-07**	-0.12	**2.16** **E** **-04**	-0.08	**9.34** **E** **-03**	0.10	**1.66** **E** **-03**

Tfh	BCL6	0.00	8.88*E*-01	-0.11	**7.79** **E** **-04**	-0.03	3.59*E*-01	-0.06	**4.91** **E** **-02**
	IL21	0.14	**9.53** **E** **-06**	0.15	**2.34** **E** **-06**	0.16	**4.56** **E** **-07**	-0.01	7.24*E*-01

Th17	STAT3	0.22	**1.13** **E** **-12**	0.09	**3.25** **E** **-03**	0.17	**1.03** **E** **-07**	0.07	**1.82** **E** **-02**
	IL17A	0.05	1.19*E*-01	0.09	**4.78** **E** **-03**	0.12	**1.04** **E** **-04**	0.01	7.77*E*-01

Treg	FOXP3	0.14	**6.97** **E** **-06**	0.15	**1.76** **E** **-06**	0.13	**2.50** **E** **-05**	0.04	2.43*E*-01
	CCR8	0.32	**4.12** **E** **-25**	0.28	**3.19** **E** **-19**	0.23	**1.86** **E** **-13**	0.05	1.10*E*-01
	TGFB1	-0.13	**4.31** **E** **-05**	-0.16	**7.90** **E** **-07**	-0.12	**9.55** **E** **-05**	-0.06	6.26*E*-02

CD8+ T	CD8A	0.00	9.85*E*-01	0.04	2.59*E*-01	0.05	1.25*E*-01	-0.02	6.23*E*-01
	CD8B	-0.03	3.52E-01	0.00	9.95*E*-01	0.04	1.98*E*-01	0.02	4.75*E*-01

T cell exhaustion	PDCD1	-0.08	**1.04** **E** **-02**	0.00	9.63*E*-01	0.02	4.87*E*-01	0.03	3.32*E*-01
	CTLA4	0.07	**2.95** **E** **-02**	0.11	**8.57** **E** **-04**	0.17	**7.80** **E** **-08**	0.05	1.56*E*-01
	LAG3	0.01	8.24*E*-01	0.10	**2.02** **E** **-03**	0.12	**8.62** **E** **-05**	0.05	9.93*E*-02
	TIM3	0.15	**1.35** **E** **-06**	0.08	**8.06** **E** **-03**	0.21	**2.01** **E** **-11**	-0.05	9.84*E*-02
	GZMB	0.03	2.80*E*-01	0.09	**2.87** **E** **-03**	0.17	**8.65** **E** **-08**	0.02	4.44*E*-01

NK cell	KIR2DL1	0.03	2.77*E*-01	0.08	**1.20** **E** **-02**	0.13	**1.96** **E** **-05**	-0.01	7.62*E*-01
	KIR3DL3	0.00	9.47*E*-01	0.04	2.64*E*-01	0.11	**3.48** **E** **-04**	-0.02	5.83*E*-01
	KIR3DL1	0.01	6.77*E*-01	0.04	2.14*E*-01	0.10	**1.47** **E** **-03**	-0.01	7.28*E*-01
	KIR3DL2	0.02	4.77*E*-01	0.06	8.03*E*-02	0.07	**2.20** **E** **-02**	-0.01	7.71*E*-01
	KIR3DL3	0.00	9.47*E*-01	0.04	2.64*E*-01	0.11	**3.48** **E** **-04**	-0.02	5.83*E*-01
	KIR2DS4	0.03	3.87*E*-01	0.07	**2.49** **E** **-02**	0.09	**4.81** **E** **-03**	-0.03	3.58*E*-01

Neutrophils	CD11b	0.04	2.60*E*-01	-0.03	3.98*E*-01	0.07	**3.23** **E** **-02**	0.06	**5.79** **E** **-02**
	CCR7	-0.01	6.45*E*-01	0.03	3.75*E*-01	-0.03	3.37*E*-01	-0.01	7.77*E*-01
	CD66b	0.00	9.75*E*-01	-0.04	1.83*E*-01	0.00	8.80*E*-01	-0.03	3.61*E*-01

M1 macrophages	NOS2	0.03	3.39*E*-01	0.06	**4.50** **E** **-02**	0.06	6.34*E*-02	-0.07	**2.10** **E** **-02**
	PTGS2	0.02	4.91*E*-01	-0.01	7.49*E*-01	0.10	**1.17** **E** **-03**	-0.12	**1.40** **E** **-04**
	IRF5	0.01	7.84*E*-01	0.11	**2.92** **E** **-04**	0.16	**6.25** **E** **-07**	0.02	5.31*E*-01

M2 macrophages	CD163	0.21	**3.01** **E** **-11**	0.23	**4.52** **E** **-13**	0.29	**3.17** **E** **-21**	0.02	6.25*E*-01
	VSIG4	0.09	**6.51** **E** **-03**	0.08	**1.25** **E** **-02**	0.16	**8.74** **E** **-07**	-0.05	1.02*E*-01
	MS4A4A	0.16	**2.37** **E** **-07**	0.13	**5.38** **E** **-05**	0.19	**7.28** **E** **-10**	-0.09	**5.86** **E** **-03**

TAM	CCL2	0.06	5.66*E*-02	0.04	1.70*E*-01	0.14	**5.57** **E** **-06**	-0.08	**1.20** **E** **-02**
	CD68	0.16	**3.26** **E** **-07**	0.11	**4.71** **E** **-04**	0.21	**1.45** **E** **-11**	-0.01	8.13*E*-01
	IL10	0.19	**1.42** **E** **-09**	0.15	**2.04** **E** **-06**	0.22	**1.38** **E** **-12**	-0.01	6.68*E*-01

Monocyte	CD86	0.14	**6.49** **E** **-06**	0.11	**5.58** **E** **-04**	0.25	**3.58** **E** **-15**	-0.05	1.28*E*-01
	CD115	-0.04	2.63*E*-01	-0.03	2.88*E*-01	0.11	**2.88** **E** **-04**	-0.07	**2.22** **E** **-02**

DC	HLA-DPB1	-0.19	**1.34** **E** **-09**	-0.17	**8.39** **E** **-08**	-0.08	**7.37** **E** **-03**	-0.03	3.46*E*-01
	HLA-DRA	0.04	2.25*E*-01	-0.02	6.17*E*-01	0.10	**1.45** **E** **-03**	-0.05	1.09*E*-01
	HLA-DQB1	-0.11	**7.12** **E** **-04**	-0.07	**1.90** **E** **-02**	-0.01	8.42*E*-01	0.04	2.44*E*-01
	HLA-DPA1	0.00	8.91*E*-01	-0.06	5.51*E*-02	0.08	**1.70** **E** **-02**	-0.08	**1.53** **E** **-02**
	CD1C	-0.14	**1.05** **E** **-05**	-0.15	**3.10** **E** **-06**	-0.15	**1.49** **E** **-06**	-0.08	**7.67** **E** **-03**
	NRP1	0.16	**5.78** **E** **-07**	0.00	8.97*E*-01	0.09	**5.97** **E** **-03**	-0.22	**4.26** **E** **-12**
	CD11c	0.03	3.55*E*-01	0.02	6.27*E*-01	0.07	**3.68** **E** **-02**	0.00	9.28*E*-01

Note: the correlation analysis was adjusted for the tumor purity. *P* values with statistical significance are shown in bold. Abbreviations: TAM: tumor-associated macrophage; Th: helper T cell; Tfh: follicular helper T cell; Treg: regulatory T cell; NK: natural killer cell; *R*: *R* value of Spearman's correlation.

**Table 3:** Correlations between HSP90s gene expression and biomarker expression of subsets of TIICs in BRAC from the GEPIA.

Types of TIICs	Biomarker	HSP90AA1		HSP90AB1		HSP90B1		TRAP1	
		*R*	*P*	*R*	*P*	*R*	*P*	*R*	*P*
B cell	CD19	-0.17	**2.20** **E** **-08**	-0.19	**5.20** **E** **-10**	-0.08	**5.50** **E** **-03**	-0.11	**4.40** **E** **-04**
	CD79A	-0.18	**9.00** **E** **-10**	-0.18	**5.30** **E** **-09**	-0.11	**2.60** **E** **-04**	-0.15	**1.30** **E** **-06**

T cell (general)	CD3D	-0.22	**9.70** **E** **-14**	-0.23	**3.00** **E** **-14**	-0.08	**5.90** **E** **-03**	-0.17	**7.60** **E** **-09**
	CD3E	-0.18	**1.90** **E** **-09**	-0.19	**3.50** **E** **-10**	-0.07	**1.70** **E** **-02**	-0.19	**2.70** **E** **-10**
	CD2	-0.12	**1.40** **E** **-04**	-0.13	**1.20** **E** **-05**	-0.03	3.60*E*-01	-0.17	**2.00** **E** **-08**

Th1	TBX21	-0.17	**2.80** **E** **-08**	-0.15	**3.50** **E** **-07**	-0.04	1.90*E*-01	-0.15	**6.40** **E** **-07**
	STAT4	-0.09	**4.50** **E** **-03**	-0.14	**3.80** **E** **-06**	-0.03	3.80*E*-01	-0.22	**1.30** **E** **-13**
	STAT1	0.29	**3.10** **E** **-22**	0.26	**1.10** **E** **-17**	0.27	**4.60** **E** **-19**	-0.01	7.20*E*-01
	TNF	-0.01	7.00*E*-01	0.05	1.20*E*-01	0.11	**5.10** **E** **-04**	0.10	**7.90** **E** **-04**
	IFNG	-0.02	5.40*E*-01	-0.02	4.80*E*-01	0.10	**1.10** **E** **-03**	-0.11	**1.90** **E** **-04**

Th2	GATA3	0.13	**1.50** **E** **-05**	0.13	**1.20** **E** **-05**	-0.11	**3.30** **E** **-04**	0.13	**1.20** **E** **-05**
	STAT6	0.10	**8.70** **E** **-04**	0.07	**2.30** **E** **-02**	-0.06	**4.20** **E** **-02**	0.10	**1.60** **E** **-03**
	IL13	-0.03	3.10*E*-01	-0.03	2.90*E*-01	0.00	9.40*E*-01	-0.07	**1.60** **E** **-02**
	STAT5A	-0.16	**1.80** **E** **-07**	-0.10	**8.40** **E** **-04**	-0.10	**6.70** **E** **-04**	0.05	9.20*E*-02

Tfh	BCL6	0.03	3.60*E*-01	-0.02	5.00*E*-01	-0.04	2.50*E*-01	-0.02	5.50*E*-01
	IL21	0.08	**1.30** **E** **-02**	0.06	**4.60** **E** **-02**	0.09	**3.30** **E** **-03**	-0.11	**2.30** **E** **-04**

Th17	STAT3	0.25	**1.60** **E** **-17**	0.20	**7.80** **E** **-11**	0.14	**6.40** **E** **-06**	0.13	**3.00** **E** **-05**
	IL17A	0.00	8.80*E*-01	0.02	5.10*E*-01	0.07	**2.20** **E** **-02**	-0.08	**6.20** **E** **-03**

Treg	FOXP3	-0.01	7.60*E*-01	-0.02	4.20*E*-01	0.02	5.10*E*-01	-0.10	**1.00** **E** **-03**
	CCR8	0.22	**1.00** **E** **-13**	0.19	**3.00** **E** **-10**	0.14	**8.10** **E** **-06**	-0.05	1.30*E*-01
	TGFB1	-0.22	**5.50** **E** **-13**	-0.24	**3.10** **E** **-16**	-0.18	**2.50** **E** **-09**	-0.14	**7.00** **E** **-06**

CD8+ T	CD8A	-0.13	**1.60** **E** **-05**	-0.13	**8.40** **E** **-06**	-0.05	1.10*E*-01	-0.17	**9.70** **E** **-09**
	CD8B	-0.16	**2.50** **E** **-07**	-0.17	**2.20** **E** **-08**	-0.06	6.80*E*-02	-0.13	**1.40** **E** **-05**

T cell exhaustion	PDCD1	-0.17	**9.30** **E** **-09**	-0.15	**1.20** **E** **-06**	-0.06	5.30*E*-02	-0.11	**1.70** **E** **-04**
	CTLA4	-0.05	1.40*E*-01	-0.05	8.20*E*-02	0.07	**2.70** **E** **-02**	-0.10	**1.10** **E** **-03**
	LAG3	-0.10	**1.60** **E** **-03**	-0.05	9.10*E*-02	0.05	9.00*E*-02	-0.03	3.00*E*-01
	TIM3	0.09	**3.70** **E** **-03**	0.03	3.30*E*-01	0.13	**1.30** **E** **-05**	-0.12	**1.30** **E** **-04**
	GZMB	-0.10	**5.70** **E** **-04**	-0.09	**3.00** **E** **-03**	0.06	**4.20** **E** **-02**	-0.11	**1.90** **E** **-04**

NK cell	KIR2DL1	-0.02	5.40*E*-01	0.00	8.90*E*-01	0.10	**6.90** **E** **-04**	-0.11	**4.70** **E** **-04**
	KIR3DL3	0.00	9.10*E*-01	0.04	2.50*E*-01	0.10	**6.50** **E** **-04**	-0.02	4.60*E*-01
	KIR3DL1	-0.08	**1.20** **E** **-02**	-0.07	**2.10** **E** **-02**	0.04	1.70*E*-01	-0.12	**4.80** **E** **-05**
	KIR3DL2	-0.06	**3.70** **E** **-02**	-0.04	2.20*E*-01	0.03	3.60*E*-01	-0.11	**2.20** **E** **-04**
	KIR3DL3	0.00	9.10*E*-01	0.04	2.50*E*-01	0.10	**6.50** **E** **-04**	-0.02	4.60*E*-01
	KIR2DS4	-0.03	3.00*E*-01	-0.03	3.50*E*-01	0.03	3.30*E*-01	-0.14	**4.90** **E** **-06**

Neutrophils	CD11b	0.00	9.40*E*-01	-0.06	**4.90** **E** **-02**	-0.01	7.20*E*-01	0.01	6.90*E*-01
	CCR7	-0.13	**9.80** **E** **-06**	-0.12	**1.20** **E** **-04**	-0.11	**2.70** **E** **-04**	-0.14	**2.10** **E** **-06**
	CD66b	0.00	1.00*E*+00	-0.03	4.10*E*-01	0.03	3.10*E*-01	-0.03	2.60*E*-01

M1 macrophages	NOS2	0.08	**1.10** **E** **-02**	0.11	**4.20** **E** **-04**	0.05	1.40*E*-01	-0.06	5.40*E*-02
	PTGS2	-0.04	1.90*E*-01	-0.06	**3.60** **E** **-02**	0.04	2.10*E*-01	-0.17	**7.60** **E** **-09**
	IRF5	0.00	9.50*E*-01	0.08	**8.20** **E** **-03**	0.14	**6.00** **E** **-06**	0.00	8.70*E*-01

M2 macrophages	CD163	0.02	4.20*E*-01	0.04	1.90*E*-01	0.15	**8.40** **E** **-07**	-0.09	**2.20** **E** **-03**
	VSIG4	0.01	7.10*E*-01	0.00	8.90*E*-01	0.08	**1.10** **E** **-02**	-0.10	**5.50** **E** **-04**
	MS4A4A	0.06	6.70*E*-02	0.02	4.90*E*-01	0.10	**1.80** **E** **-03**	-0.15	**3.50** **E** **-07**

TAM	CCL2	-0.05	1.20*E*-01	-0.07	**3.40** **E** **-02**	0.08	**1.20** **E** **-02**	-0.15	**4.50** **E** **-07**
	CD68	0.15	**3.40** **E** **-07**	0.10	**1.50** **E** **-03**	0.08	**1.20** **E** **-02**	-0.07	**1.90** **E** **-02**
	IL10	0.14	**3.00** **E** **-06**	0.11	**1.90** **E** **-04**	0.14	**7.80** **E** **-06**	-0.09	**4.10** **E** **-03**

Monocyte	CD86	0.07	**3.20** **E** **-02**	0.03	3.50*E*-01	0.15	**3.60** **E** **-07**	-0.14	**2.00** **E** **-06**
	CD115	-0.09	**3.00** **E** **-03**	-0.08	**8.40** **E** **-03**	0.03	3.50*E*-01	-0.13	**2.30** **E** **-05**

DC	HLA-DPB1	-0.25	**2.20** **E** **-16**	-0.26	**7.70** **E** **-18**	-0.14	**1.80** **E** **-06**	-0.16	**5.70** **E** **-08**
	HLA-DRA	-0.08	**9.60** **E** **-03**	-0.13	**8.70** **E** **-06**	-0.01	7.20*E*-01	-0.16	**6.80** **E** **-08**
	HLA-DQB1	-0.20	**6.80** **E** **-11**	-0.20	**1.40** **E** **-11**	-0.06	**5.00** **E** **-02**	-0.09	**4.30** **E** **-03**
	HLA-DPA1	-0.09	**5.20** **E** **-03**	-0.14	**2.80** **E** **-06**	-0.02	6.10*E*-01	-0.16	**4.60** **E** **-08**
	CD1C	-0.24	**6.90** **E** **-16**	-0.24	**2.80** **E** **-16**	-0.20	**1.90** **E** **-11**	-0.18	**3.20** **E** **-09**
	NRP1	0.12	**8.30** **E** **-05**	0.01	8.00*E*-01	0.02	5.10*E*-01	-0.20	**1.70** **E** **-11**
	CD11c	-0.07	**2.80** **E** **-02**	-0.09	**4.20** **E** **-03**	-0.03	3.20*E*-01	-0.08	**5.90** **E** **-03**

Note: the same as Table 2.

## Data Availability

The prognostic significance of the HSP90AA1, HSP90AB1, HSP90B1, and TRAP1 expression in BRAC patients with different clinical parameters is shown in Supplementary Tables 1, 2, 3, and 4, respectively. All the data that support the findings of this study are publicly available in https://www.oncomine.org/, https://ualcan.path.uab.edu/, https://kmplot.com/, https://cistrome.shinyapps.io/timer/, https://gepia.cancer-pku.cn/, https://genemania.org/, and https://david.ncifcrf.gov/.

## Supplementary Materials

Supplementary Materials The prognostic significance of the HSP90AA1, HSP90AB1, HSP90B1, and TRAP1 expression in BRAC patients with different clinical parameters is shown in Supplementary Tables 1, 2, 3, and 4, respectively.
