# Supplementary material for: Heat Shock Protein 90 Family Isoforms as Prognostic Biomarkers and Their Correlations with Immune Infiltration in Breast Cancer
Source: Biomed Res Int. 2020 Oct 21;2020:2148253. doi: 10.1155/2020/2148253 (PMC7596464; doi:10.1155/2020/2148253)
Supplement: Supplementary Materials — The prognostic significance of the HSP90AA1, HSP90AB1, HSP90B1, and TRAP1 expression in BRAC patients with different clinical parameters is shown in Supplementary Tables 1, 2, 3, and 4, respectively. [file 2148253.f1.zip › 2148253.f1.docx]

Supplementary Table 1: Prognostic significance of HSP90AA1 expression in BRAC patients with different clinical parameters.

|  | Overall survivals | | | Release-free survivals | | |
| --- | --- | --- | --- | --- | --- | --- |
|  | N | HR (95% CI) | *P* value | N | HR (95% CI) | *P* value |
| ER status |  |  |  |  |  |  |
| ER+ | 548 | 1.08(0.76-1.54) | 6.60E-01 | 801 | 1.39(1.18-1.64) | **6.80E-05** |
| ER− | 251 | 1.5(0.95-2.39) | 8.20E-02 | 801 | 1.19(0.95-1.49) | 1.30E-01 |
| PR status |  |  |  |  |  |  |
| PR+ | 83 | 0.87(0.23-3.25) | 8.30E-01 | 589 | 1.45(1.02-2.06) | **3.50E-02** |
| PR− | 89 | 0.94(0.37-2.37) | 9.00E-01 | 549 | 1.03(0.77-1.38) | 8.20E-01 |
| HER2 status |  |  |  |  |  |  |
| HER2+ | 129 | 0.58(0.28-1.21) | 1.40E-01 | 252 | 0.97(0.63-1.5) | 9.00E-01 |
| HER2− | 130 | 0.84(0.35-2.01) | 6.90E-01 | 800 | 1.35(1.03-1.75) | **2.70E-02** |
| Intrinsic subtypes |  |  |  |  |  |  |
| Basal | 879 | 1.69(1.02-2.8) | **3.80E-02** | 618 | 1.51(1.17-1.95) | **1.30E-03** |
| Luminal A | 611 | 1.22(0.86-1.74) | 2.60E-01 | 1933 | 1.67(1.4-1.99) | **4.80E-09** |
| Luminal B | 433 | 0.88(0.61-1.28) | 5.20E-01 | 1149 | 1.28(1.05-1.55) | **1.30E-02** |
| HER2 enriched | 117 | 1.3(0.68-2.49) | 4.20E-01 | 251 | 1.47(1-2.16) | **4.90E-02** |
| Lymph node status | |  |  |  |  |  |
| + | 313 | 0.91(0.62-1.34) | 6.40E-01 | 1133 | 1.24(1.02-1.51) | **3.00E-02** |
| − | 594 | 1.6(1.1-2.33) | **1.30E-02** | 2020 | 1.6(1.1-2.33) | **1.30E-02** |
| Grade |  |  |  |  |  |  |
| 1 | 161 | 1.01(0.42-2.44) | 9.80E-01 | 345 | 1.14(0.68-1.91) | 6.30E-01 |
| 2 | 387 | 1.47(0.95-2.26) | 8.00E-02 | 901 | 1.65(1.29-2.11) | **5.10E-05** |
| 3 | 503 | 1.12(0.81-1.56) | 4.90E-01 | 903 | 1.17(0.94-1.45) | 1.60E-01 |
| Stage |  |  |  |  |  |  |
| 1 | 180 | 1.16(0.43-3.1) | 7.70E-01 | 165 | 2.34(0.58-9.5) 4.5 | 2.20E-01 |
| 2 | 619 | 2.43(1.44-4.09) | **5.70E-04** | 554 | 1.65(0.83-3.28) | 1.50E-01 |
| 3 | 247 | 1.34(0.74-2.42) | 3.30E-01 | 212 | 1.37(0.7-2.69) | 3.60E-01 |
| 4 | 20 | 0.7(0.24-2.04) | 5.20E-01 | - | - | - |

*Note: P*<0.05 is recognized as statistical significance, and these *P* values are shown in bold. *Abbreviations:* HR, hazard ratio; CI, confidence interval.
