# Supplementary material for: Heat Shock Protein 90 Family Isoforms as Prognostic Biomarkers and Their Correlations with Immune Infiltration in Breast Cancer
Source: Biomed Res Int. 2020 Oct 21;2020:2148253. doi: 10.1155/2020/2148253 (PMC7596464; doi:10.1155/2020/2148253)
Supplement: Supplementary Materials — The prognostic significance of the HSP90AA1, HSP90AB1, HSP90B1, and TRAP1 expression in BRAC patients with different clinical parameters is shown in Supplementary Tables 1, 2, 3, and 4, respectively. [file 2148253.f1.zip › 2148253.f2.docx]

Supplementary Table 2: Prognostic significance of HSP90AB1 expression in BRAC patients with different clinical parameters.

|  | Overall survivals | | | Release-free survivals | | |  |
| --- | --- | --- | --- | --- | --- | --- | --- |
|  | N | HR (95% CI) | *P* value | N | HR (95% CI) | *P* value |  |
| ER status |  |  |  |  |  |  |  |
| ER+ | 548 | 1.18(0.82-1.68) | 3.70E-01 | 2061 | 1.29(1.1-1.52) | **2.00E-03** |  |
| ER− | 251 | 0.61(0.38-0.97) | **3.30E-02** | 801 | 0.89(0.71-1.12) | 3.10E-01 |  |
| PR status |  |  |  |  |  |  |  |
| PR+ | 83 | 0.35(0.07-1.68) | 1.70E-01 | 589 | 1.39(0.98-1.98) | 6.30E-02 |  |
| PR− | 89 | 0.43(0.16-1.16) | 8.60E-02 | 549 | 1.26(0.94-1.69) | 1.20E-01 |  |
| HER2 status |  |  |  |  |  |  |  |
| HER2+ | 129 | 0.56(0.27-1.15) | 1.10E-01 | 252 | 0.61(0.39-0.94) | **2.40E-02** |  |
| HER2− | 130 | 0.44(0.17-1.14) | 8.30E-02 | 800 | 1.33(1.03-1.74) | **3.10E-02** |  |
| Intrinsic subtypes |  |  |  |  |  |  |  |
| Basal | 879 | 0.5(0.3-0.82) | **5.30E-03** | 618 | 0.99(0.77-1.28) | 9.60E-01 |  |
| Luminal A | 611 | 1.22(0.85-1.74) | 2.80E-01 | 1933 | 1.27(1.07-1.51) | **5.80E-03** |  |
| Luminal B | 433 | 1.05(0.72-1.52) | 8.20E-01 | 1149 | 1.12(0.92-1.35) | 2.70E-01 |  |
| HER2 enriched | 117 | 0.69(0.36-1.32) | 2.60E-01 | 251 | 1.03(0.7-1.51) | 8.80E-01 |  |
| Lymph node status | |  |  |  |  |  |  |
| + | 313 | 0.96(0.64-1.39) | 7.60E-01 | 1133 | 1.12(0.92-1.36) | 2.70E-01 |  |
| − | 594 | 1.21(0.84-1.76) | 3.10E-01 | 2020 | 1.32(1.11-1.56) | **1.40E-03** |  |
| Grade |  |  |  |  |  |  |  |
| 1 | 161 | 1.36(0.53-3.49) | 5.30E-01 | 345 | 1.54(0.91-2.61) | 1.00E-01 |  |
| 2 | 387 | 1.13(0.74-1.74) | 5.70E-01 | 901 | 1.21(0.95-1.54) | 1.20E-01 |  |
| 3 | 503 | 0.79(0.57-1.09) | 1.50E-01 | 903 | 1.04(0.84-1.3) | 7.00E-01 |  |
| Stage |  |  |  |  |  |  |  |
| 1 | 180 | 0.84(0.31-2.27) | 7.30E-01 | 165 | 2.78(0.68-11.3) 3.5 | 1.40E-01 |  |
| 2 | 619 | 1.32(0.81-2.14) | 2.70E-01 | 554 | 1.18(0.6-2.33) | 6.20E-01 |  |
| 3 | 247 | 1.51(0.83-2.75) | 1.70E-01 | 212 | 1.3(0.66-2.56) | 4.40E-01 |  |
| 4 | 20 | 1.55(0.52-4.6) 4.5 | 4.30E-01 | - | - | - |  |

*Note: P*<0.05 is recognized as statistical significance, and these *P* values are shown in bold. *Abbreviations:* HR, hazard ratio; CI, confidence interval.
