# Supplementary material for: Heat Shock Protein 90 Family Isoforms as Prognostic Biomarkers and Their Correlations with Immune Infiltration in Breast Cancer
Source: Biomed Res Int. 2020 Oct 21;2020:2148253. doi: 10.1155/2020/2148253 (PMC7596464; doi:10.1155/2020/2148253)
Supplement: Supplementary Materials — The prognostic significance of the HSP90AA1, HSP90AB1, HSP90B1, and TRAP1 expression in BRAC patients with different clinical parameters is shown in Supplementary Tables 1, 2, 3, and 4, respectively. [file 2148253.f1.zip › 2148253.f3.docx]

Supplementary Table 3: Prognostic significance of HSP90B1 expression in BRAC patients with different clinical parameters.

|  | Overall survivals | | | Release-free survivals | | |  |
| --- | --- | --- | --- | --- | --- | --- | --- |
|  | N | HR (95% CI) | *P* value | N | HR (95% CI) | *P* value |  |
| ER status |  |  |  |  |  |  |  |
| ER+ | 548 | 1.13(0.8-1.61) | 4.80E-01 | 2061 | 1.24(1.06-1.46) | **8.80E-03** |  |
| ER− | 251 | 1.09(0.69-1.71) | 7.20E-01 | 801 | 0.96(0.77-1.2) | 7.30E-01 |  |
| PR status |  |  |  |  |  |  |  |
| PR+ | 83 | 1.19(0.32-4.46) | 1.70E-01 | 589 | 1.39(0.98-1.97) | 6.40E-02 |  |
| PR− | 89 | 1.11(0.44-2.79) | 8.30E-01 | 549 | 1.02(0.76-1.36) | 9.10E-01 |  |
| HER2 status |  |  |  |  |  |  |  |
| HER2+ | 129 | 1.37(0.67-2.82) | 3.80E-01 | 252 | 1.33(0.86-2.06) | 2.00E-01 |  |
| HER2− | 130 | 2.15(0.83-5.56) | 1.06E+00 | 800 | 1.3(1-1.68) | 5.20E-02 |  |
| Intrinsic subtypes |  |  |  |  |  |  |  |
| Basal | 879 | 0.76(0.46-1.24) | 2.70E-01 | 618 | 0.86(0.67-1.1) | 2.30E-01 |  |
| Luminal A | 611 | 1.26(0.88-1.79) | 2.00E-01 | 1933 | 1.24(1.04-1.46) | **1.50E-02** |  |
| Luminal B | 433 | 0.97(0.67-1.41) | 8.80E-01 | 1149 | 1.19(0.99-1.45) | 6.80E-02 |  |
| HER2 enriched | 117 | 0.82(0.43-1.57) | 5.60E-01 | 251 | 1.1(0.75-1.61) | 6.40E-01 |  |
| Lymph node status | |  |  |  |  |  |  |
| + | 313 | 0.97(0.66-1.42) | 8.60E-01 | 1133 | 1.29(1.06-1.57) | **1.20E-02** |  |
| − | 594 | 1.1(0.76-1.6) | 6.00E-01 | 2020 | 1.07(0.91-1.27) | 4.00E-01 |  |
| Grade |  |  |  |  |  |  |  |
| 1 | 161 | 1.28(0.53-3.11) | 5.80E-01 | 345 | 1.29(0.77-2.16) | 3.40E-01 |  |
| 2 | 387 | 1.25(0.82-1.92) | 3.10E-01 | 901 | 0.92(0.72-1.17) | 5.00E-01 |  |
| 3 | 503 | 0.96(0.69-1.33) | 7.90E-01 | 903 | 1.08(0.86-1.34) | 5.10E-01 |  |
| Stage |  |  |  |  |  |  |  |
| 1 | 180 | 0.7(0.26-1.9) | 4.80E-01 | 165 | 0.24(0.05-1.14) | 5.10E-02 |  |
| 2 | 619 | 1.03(0.63-1.67) | 9.10E-01 | 554 | 0.78(0.4-1.54) | 4.70E-01 |  |
| 3 | 247 | 0.54(0.29-0.99) | **4.30E-02** | 212 | 0.66(0.34-1.31) | 2.30E-01 |  |
| 4 | 20 | 0.26(0.08-0.86) | **2.00E-02** | - | - | - |  |

*Note: P*<0.05 is recognized as statistical significance, and these *P* values are shown in bold. *Abbreviations:* HR, hazard ratio; CI, confidence interval.
