# Supplementary material for: Heat Shock Protein 90 Family Isoforms as Prognostic Biomarkers and Their Correlations with Immune Infiltration in Breast Cancer
Source: Biomed Res Int. 2020 Oct 21;2020:2148253. doi: 10.1155/2020/2148253 (PMC7596464; doi:10.1155/2020/2148253)
Supplement: Supplementary Materials — The prognostic significance of the HSP90AA1, HSP90AB1, HSP90B1, and TRAP1 expression in BRAC patients with different clinical parameters is shown in Supplementary Tables 1, 2, 3, and 4, respectively. [file 2148253.f1.zip › 2148253.f4.docx]

Supplementary Table 4: Prognostic significance of TRAP1 expression in BRAC patients with different clinical parameters.

|  | Overall survivals | | | Release-free survivals | | |  |
| --- | --- | --- | --- | --- | --- | --- | --- |
|  | N | HR (95% CI) | *P* value | N | HR (95% CI) | *P* value |  |
| ER status |  |  |  |  |  |  |  |
| ER+ | 548 | 0.85(0.6-1.22) | 3.90E-01 | 2061 | 0.93(0.79-1.1) | 4.10E-01 |  |
| ER− | 251 | 0.72(0.45-1.15) | 1.70E-01 | 801 | 0.93(0.74-1.16) | 5.10E-01 |  |
| PR status |  |  |  |  |  |  |  |
| PR+ | 83 | 0.26(0.05-1.27) | 7.40E-02 | 589 | 0.94(0.66-1.33) | 7.20E-01 |  |
| PR− | 89 | 1.42(0.56-3.62) | 4.50E-01 | 549 | 0.93(0.7-1.25) | 6.40E-01 |  |
| HER2 status |  |  |  |  |  |  |  |
| HER2+ | 129 | 0.7(0.34-1.46) | 3.40E-01 | 252 | 1.31(0.84-2.02) | 2.30E-01 |  |
| HER2− | 130 | 0.58(0.22-1.52) | 2.60E-01 | 800 | 0.77(0.59-1) | **4.60E-02** |  |
| Intrinsic subtypes |  |  |  |  |  |  |  |
| Basal | 879 | 1.29(0.79-2.12) | 3.10E-01 | 618 | 0.83(0.64-1.07) | 1.40E-01 |  |
| Luminal A | 611 | 0.72(0.5-1.03) | 7.20E-02 | 1933 | 0.66(0.55-0.78) | **1.90E-06** |  |
| Luminal B | 433 | 0.99(0.68-1.44) | 9.60E-01 | 1149 | 0.79(0.65-0.96) | 1.70E-02 |  |
| HER2 enriched | 117 | 0.76(0.39-1.45) | 4.00E-01 | 251 | 0.84(0.57-1.23) | 3.70E-01 |  |
| Lymph node status | |  |  |  |  |  |  |
| + | 313 | 0.94(0.64-1.39) | 7.60E-01 | 1133 | 0.74(0.61-0.9) | **2.40E-03** |  |
| − | 594 | 0.73(0.5-1.06) | 9.70E-02 | 2020 | 0.99(0.84-1.17) | 9.20E-01 |  |
| Grade |  |  |  |  |  |  |  |
| 1 | 161 | 0.82(0.32-2.14) | 6.90E-01 | 345 | 0.79(0.47-1.34) | 3.80E-01 |  |
| 2 | 387 | 0.97(0.63-1.49) | 8.90E-01 | 901 | 1.03(0.81-1.31) | 8.00E-01 |  |
| 3 | 503 | 0.99(0.71-1.37) | 9.30E-01 | 903 | 1.1(0.89-1.37) | 3.80E-01 |  |
| Stage |  |  |  |  |  |  |  |
| 1 | 180 | 0.73(0.27-1.97) | 5.30E-01 | 165 | 1.03(0.27-3.84) | 9.70E-01 |  |
| 2 | 619 | 0.85(0.52-1.39) | 5.20E-01 | 554 | 0.52(0.26-1.06) | 6.70E-02 |  |
| 3 | 247 | 0.56(0.3-1.03) | 5.70E-02 | 212 | 0.96(0.49-1.88) | 9.00E-01 |  |
| 4 | 20 | 1.12(0.38-3.26) | 8.40E-01 | - | - | - |  |

*Note: P*<0.05 is recognized as statistical significance, and these *P* values are shown in bold. *Abbreviations:* HR, hazard ratio; CI, confidence interval.
